# Tissue-Specific 5′ Heterogeneity of PPARα Transcripts and Their Differential Regulation by Leptin

**DOI:** 10.1371/journal.pone.0067483

**Published:** 2013-06-25

**Authors:** Emma S. Garratt, Mark H. Vickers, Peter D. Gluckman, Mark A. Hanson, Graham C. Burdge, Karen A. Lillycrop

**Affiliations:** 1 Academic Unit of Human Development and Health, Faculty of Medicine, University of Southampton, Southampton, United Kingdom; 2 Liggins Institute and the National Research Centre for Growth and Development, University of Auckland, Auckland, New Zealand; 3 Centre for Biological Sciences, Faculty of Natural and Environmental Sciences, University of Southampton, Southampton, United Kingdom; Nihon University School of Medicine, Japan

## Abstract

The genes encoding nuclear receptors comprise multiple 5′untranslated exons, which give rise to several transcripts encoding the same protein, allowing tissue-specific regulation of expression. Both human and mouse peroxisome proliferator activated receptor (PPAR) α genes have multiple promoters, although their function is unknown. Here we have characterised the rat PPARα promoter region and have identified three alternative PPARα transcripts, which have different transcription start sites owing to the utilisation of distinct first exons. Moreover these alternative PPARα transcripts were differentially expressed between adipose tissue and liver. We show that while the major adipose (P1) and liver (P2) transcripts were both induced by dexamethasone, they were differentially regulated by the PPARα agonist, clofibric acid, and leptin. Leptin had no effect on the adipose-specific P1 transcript, but induced liver-specific P2 promoter activity via a STAT3/Sp1 mechanism. Moreover in Wistar rats, leptin treatment between postnatal day 3–13 led to an increase in P2 but not P1 transcription in adipose tissue which was sustained into adulthood. This suggests that the expression of the alternative PPARα transcripts are in part programmed by early life exposure to leptin leading to persistent change in adipose tissue fatty acid metabolism through specific activation of a quiescent PPARα promoter. Such complexity in the regulation of PPARα may allow the expression of PPARα to be finely regulated in response to environmental factors.

## Introduction

PPARα is a ligand-activated transcription factor, which belongs to the nuclear hormone receptor superfamily [1], [2]. PPARα plays a major role in lipid homeostasis by regulating the transcription of genes that encode the rate limiting enzymes in β oxidation, namely carnitine palmitoyl transferase (CPT-1) and acyl CoA oxidase (AOX)[3]–[5]. Targeted disruption of the PPARα gene in mice leads to lipid accumulation in the liver, impaired insulin secretion during fasting [6], increased adipose tissue mass and an increased incidence of liver tumours [7], [8]. Consistent with these findings, agonists of PPARα have been used as effective hypolipidemic drugs [9].

PPARα is mainly expressed in tissues with high rates of fatty acid β oxidation such as liver, skeletal muscle, brown fat, heart, and kidneys[10]; [11]. Its expression is known to be regulated through the action of glucocorticoids [12], by HNF4, a major regulator of gluconeogenesis, [13], and by PPARα itself [14], [15]. Adenoviral induced hyperleptinemia, which causes a rapid loss of body fat without a rise in plasma FFA or ketone bodies, has also been shown to increase the expression of PPARα and its target genes in white adipose tissue, a tissue where PPARα is not normally expressed. Conversely, the expression of PPARγ2 and its associated genes involved in lipogenesis were reduced. However, the effects of hyperleptinemia were transient and two months after the concentration of leptin returned to normal, levels of PPARα expression decreased in adipose tissue and fat levels were regained [16]–[19]. This transient transformation of adipocytes from fat storing cells into fat burning cells via the induction of PPARα expression might suggest a novel approach for the treatment of obesity and a potential target for weight reduction.

There is also evidence that PPARα gene transcription can be programmed by environmental factors in early life [20]. For example the expression of PPARα is increased in the liver of offspring born to dams fed a protein restricted (PR) diet during pregnancy. The increase in PPARα expression in the PR offspring is accompanied by the increased expression of its target gene acyl-CoA oxidase (AOX) and an increase in levels of fatty acid beta-oxidation[20 21]. In contrast, a 70% global dietary restriction during pregnancy induces a persistent decrease in PPARα expression in the liver of the adult offspring suggesting that different nutritional challenges during pregnancy induce distinct long term effects on PPARα expression [22]. In addition, there is evidence in the rat that PPARα expression is programmed by neonatal leptin exposure [22], [23]. Neonatal leptin administration which reverses the phenotypic effects of maternal under nutrition by slowing neonatal weight gain, normalizing caloric intake and locomotor activity, induced a persistent increase in hepatic PPARα expression [22]. This is in contrast to the effects of hyperleptinaemia on PPARα expression in adipose tissue of adult rats where the increase in PPARα expression was not sustained after leptin administration was discontinued, suggesting that the timing of leptin exposure may determine the longevity of the response.

To date, little is known about of the molecular factors that mediate the tissue-specific regulation of PPARα, or its regulation by perinatal factors or leptin. The genes encoding nuclear receptors frequently comprise multiple 5′untranslated exons giving rise to transcripts, which are expressed differentially between tissues. The human [24], mouse [25], and rat [26] PPARα gene promoters have only been partially characterised. The human PPARα gene is composed of 12 exons and generates 7 mRNA variants with different 5′UTR exons. The 5′UTR of the human PPARα gene contains 7 exons (exons A, 1A, B, 1B, 2A, 2B and the 5′end of exon 3), while the coding exons are derived from the remainder of exon 3 and exons 4–8[27]–[29]. The mouse PPARα gene is composed of 9 exons and generates 3 transcripts. Four exons comprise the 5′UTR (exon 1a, 1b, 2 and the 5′end of exon 3), the coding exons, like the human orthologue, are composed of the 3′end of exon 3 and exons 4–8 [25]. The rat PPARα gene is comprised of 8 exons with 1 transcript reported to date. Three exons comprise the 5′UTR exons, exon 1, 2 and the 5′ portion of exon 3 [26]. Because of the complexities of the human and mouse genes, we hypothesised that the rat PPARα gene might also exhibit heterogeneity in the 5′UTR leading to the synthesis of multiple transcripts. We have, therefore, characterised the promoter structure of the rat PPARα gene, and determined the pattern of expression between tissues and the extent to which alternative transcripts are differentially regulated by leptin. We show that rat PPARα gene gives rise to three PPARα mRNA variants, which differ from each other at the 5′ end owing to the presence of unique first exons. The alternative PPARα transcripts were differentially expressed between adipose tissue and liver. We show that while the major adipose (P1) and liver (P2) transcripts were both induced by dexamethasone, they were differentially regulated by clofibric acid and leptin. Leptin had no effect on the adipose specific P1 transcript, but induced the liver specific P2 promoter activity via a STAT3/Sp1 mechanism. Moreover, consistent with leptin regulation of transcription from the P2 promoter in vitro, neonatal leptin treatment led to a persistent increase in transcription from the P2 promoter and not the P1 promoter in adipose tissue. Such 5′ heterogeneity and complexity of regulation of PPARα may provide additional layers of control by which PPARα expression can be intricately regulated in response to hormones and early life environment in a tissue specific manner.

## Materials and Methods

### Ethics Statement

All animal work was approved by the Animal Ethics Committee of the University of Auckland (Approval N856) and complied with the New Zealand Code of Ethical Conduct for the care and use of animals for scientific purposes (Animal Welfare Act, 1999).

### Animal Methods

A detailed description of the study design has been published previously [23]. Briefly, virgin Wistar rats (age 100±5 days) were time-mated and fed a standard rat chow fed *ad libitum* throughout gestation. Litter size was adjusted to 8 pups at birth to ensure standardised nutrition until weaning. At postnatal day 3, female pups were randomized to receive either saline or recombinant rat leptin (rat leptin from Protein Laboratories, Rehovot, Israel) (2.5 µg/g/day) for 10 days by subcutaneous injection (n = 16 per group). Dams were fed *ad libitum* until offspring were weaned on day 22. Saline or leptin-treated offspring were weaned onto standard rat chow. This produced 8 groups of female rats (n = 8 per group). On postnatal day 170, rats were fasted overnight, and killed by halothane anaesthesia followed by decapitation. Liver and retroperitoneal adipose tissue was removed immediately, frozen in liquid nitrogen and stored at −80°C.

### Analysis of mRNA Expression

Total PPARα, P1 and P2 PPARα, AOX and carnitine palmitoyltransferase (CPT)-1 mRNA concentrations were determined by real time RTPCR [30], [31]. Briefly, total RNA was isolated from cells using TRIZOL reagent (InVitrogen, Paisley, Scotland, UK), and 1 µg was used as a template to prepare cDNA using 100 U Moloney-Murine Leukemia Virus reverse transcriptase. cDNA was amplified using real time PCR primers specific to total PPARα, P1 and P2 transcripts, AOX and CPT-1 (Table 1). The reaction was performed in a total volume of 25 µl with SYBR® Green Jumpstart Ready Mix (Sigma, Poole, Dorset, UK) as described by the manufacturer. Samples were analyzed in duplicate and Ct values were normalised to ribosomal 18S RNA using the ΔΔCt method [30].

**Table 1:** Primer sequences used in the measurements of mRNA expression by real time RT PCR.

	Forward primer	Reverse primer
PPARα	CGGGTCATACTCGCAGGAAAG	TGGCAGCAGTGGAAGAATCG
CPT-1	ACCACTGGCCGAATGTCAAG	AGCGAGTAGCGCATGGTCAT
AOX	CCAATCACGCAATAGTTCTGG	CGCTGTATCGTATGGCGAT
PPARα P1	ATGAGCTCAGCAGCGTCCTGAGGCGTT	ATAAGCTTACCTGAGGCTGCGCTCCG
PPARα P2	ATGAGCTCAGCAGCGTCCTGAGGCGTT	ATAAGCTTGTGCCCTTCCTAGCGTGT
18S	GTAACCCGTTGAACCCCATT	CCATCCAATCGGTAGTAGTAGCG

PPARα, peroxisomal proliferator-activated receptor-α; CPT-1, carnitine:palmitoyl transferase-1; AOX, acyl-CoA oxidase;.

### 5′ RNA Ligase Mediated Rapid Amplification of cDNA Ends (5′ RLM RACE)

RNA was amplified using a 5′ RNA Ligase Mediated Rapid Amplification of cDNA Ends (RLM RACE) kit (Ambion) according to manufacturer’s instructions. Briefly, 10 µg of total RNA from liver and adipose tissue was incubated with calf intestinal alkaline phosphatase to remove free 5′ phosphates, the 5′ CAP structure was then removed from the RNA with tobacco acid pyrophosphatase (TAP) and the TAP treated RNA ligated to the 5′ RACE adapter and reverse transcribed into cDNA using random hexamers. To amplify the 5′ region of PPARα mRNA, generated from the 5′RLM RACE, nested gene specific reverse primers for PPARα were designed (inner primer 5′ TGACTGAGGAGGGGCTGGAA 3′; outer gene specific primer 5′ AGCCTTCACATGCGTGGACT 3) and used with the nested forward 5′ RACE adaptor primers provided by the kit. Cycling conditions for both outer and nested PCR were initial denaturation of 94°C 3 minutes, followed by 35 cycles of 94°C 30 s, anneal at 60°C 30 sec, and extension at 72°C 1 minute 30 sec. Resulting PCR products were purified, cloned into pGem T-Easy (Promega) and sequenced. The sequence of the transcripts P1–3 was confirmed by sequencing multiple clones from liver and adipose tissue.

### DNA Cloning

100 ng rat genomic DNA was used as a template for PCR amplification with primers designed to amplify 1–1.5 Kb of upstream sequence from the transcription start site of the P1, P2 and P3 promoters (P1 and P2 forward primer: 5′ATGAGCTCAGCAGCGTCCTGAGGCGTT 3′; P1 reverse primer 5′ ATAAGCTTACCTGAGGCTGCGCTCCG 3′; P2 reverse primer 5′ ATAA.

GCTTGTGCCCTTCCTAGCGTGT 3′; P3 forward: 5′ ATAAGCTTGGAGTCTTCCTTCTGGTT 3′; P3 reverse 5′ ATACTCGAGTCTGCGTGGGTGTCTAAT 3′). All primers contained either a SacI or XhoI restriction site at the 5′ end of the forward primer, and a HindIII restriction site at the 5′ end of the reverse primer to allow cloning of PCR fragments into the pGL3 Basic reporter vector (Promega). Cycling conditions for PCR were as follows; initial denaturation of 94°C 2 minutes, followed by 40 cycles of 95°C 45 s, anneal at a 60°C 45 sec, and extension at 72°C 5 minutes. Resulting PCR products were gel extracted, cloned into the pGL3 Basic reporter vector (Promega) and sequenced to confirm the presence of the insert. The Quik change method of mutagenesis (Stratagene) was used to create a P2 promoter construct with a mutated Sp1 site. Two complementary primers were designed using the QuikChange® Primer Design Program (Stratagene, Texas, USA) to change the Sp1 consensus sequence GGGCGG to an EcoR1 restriction site GAATTC (Forward primer 5′ GCCTCAGGTGCCCAGGAATTCGAGGGCACGCGCGAGG 3′ and reverse complement 5′ CCTCGCGCGTGCCCTCGAATTCCTGGGCACCTGAGGC 3′). The primers (0.4 µM) were then annealed to 30 ng P2- pGL3 DNA and extended using Pfu polymerase. Cycling conditions for PCR were as follows; initial denaturation of 95°C 2 minutes, followed by 18 cycles of 95°C 30 s, anneal at a 60°C 1 min, and extension at 72°C 4 minutes, followed by a final extension at 72°C for 5 minutes. Resulting PCR products were digested with the restriction enzyme DpnI at 37°C for 1 hour and the product transformed into DH5α E.Coli. Clones were cut with EcoR1 and sequenced to confirm the presence of the mutation.

### Cell Culture and Transfections

The human hepatoma cell line HepG2 (ECACC- Sigma Aldrich) was cultured in DMEM supplemented with 10% fetal bovine serum, 2 mM glutamine, 10 u/ml penicillin and 100 ug/ml streptomycin. HepG2 cells were transfected using calcium phosphate [31], [32], clofibric acid, dexamethasone, the Stat3 inhibitor PpYLKTK-mts and leptin were added immediately after transfection at the stated concentrations and luciferase activity measured 24 hrs later using the Luciferase Assay System (Promega). All transfections were performed in triplicate and values are expressed as luciferase activity per mg of protein.

### Statistical Analysis

Statistical comparisons of mRNA expression and methylation levels between treatments relative to the untreated control were carried out using a Students unpaired t test. Statistical comparisons of luciferase activity levels between promoter constructs and treatments were determined by ANOVA followed by Bonferoni’s post hoc analysis. All values are plotted as the mean ±SEM.

## Results

### 5′RACE Analysis Identifies Distinct Tissue Specific 5′leader Sequences in Rat PPARα mRNA

**Figure 1:**
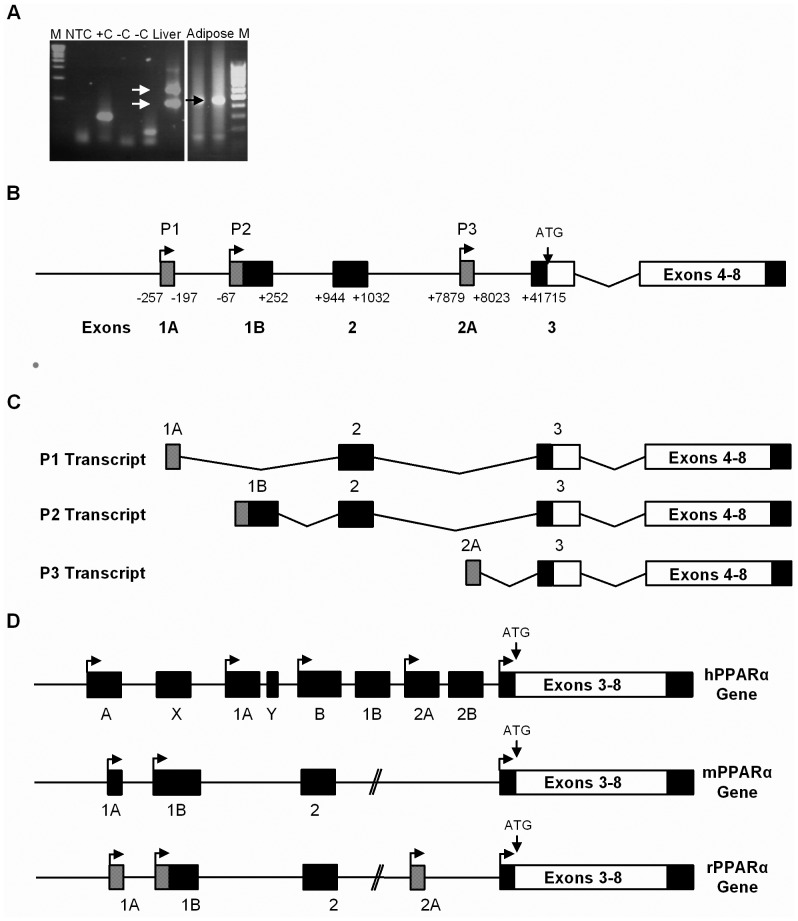
Tissue specific 5′ heterogeneity of PPARα transcripts. A) 5′ RLM RACE PCR indicates 2 major PPARα transcripts in liver and one in adipose. M, DNA ladder; NTC, no template control; +C, RACE positive control; -C, negative control; -C, -TAP control; liver, liver cDNA; adipose, adipose cDNA; M, DNA ladder. B) A schematic diagram showing the genomic organisation of the rat PPARα gene. Location of PPARα 5′UTR exons on the genomic sequence are shown (non-coding exons, black; coding exons, white and the updated exons, grey). All exon positions are indicated relative to the Ensembl transcription start site. Ensembl exon 3 which contains the translation ATG start codon is present in all transcripts. C) Diagram showing the adipose specific transcript (P1) and liver specific transcripts (P2 and P3). D) Comparison of the Rat PPARα 5′UTR with the 5′UTR of the human and mouse PPARα genes. Non-coding 5′UTR exons are shown in black, coding exons in white and newly identified non coding exons in grey.

To investigate tissue specific 5′heterogeneity of rat PPARα mRNA, 5′RLM RACE PCR analysis was conducted on total RNA from adipose and liver tissue from adult Wistar rats. 5′RLM RACE revealed 2 different sized PCR products from the liver (723 bp and 463 bp) but only 1 from adipose tissue (465 bp) (Figure. 1A). The PCR products were cloned into the pGEM T-easy vector and the resulting clones sequenced and compared to the published genomic sequence of rat PPARα (Ensemble gene ID ENSRNOG00000021463) to identify the genomic location of the exons. The 3 transcripts differed from each other at the 5′ end, and comparisons to the genomic sequence showed that they had different transcription start sites owing to the presence of unique first exons. The PPARα transcripts were subsequently termed P1–3 and a schematic diagram indicating the genomic location of the PPARα 5′UTR exons and the organisation of the alternative 5′UTRs is shown in Figure 1b.

Five exons were found to encode the PPARα 5′UTR covering a genomic region of 42010 bps. Exons were named according to their genomic location. Exons 1A and 2A were novel, one exon was a modified version of exon 1 (now termed 1B), and the remainder were exactly as identified by Ensembl (exons 2 and 3). The 5′UTR of the adipose specific transcript (P1) is encoded by exons 1A, 2 and 3. The transcription start site for this transcript is 257 bp upstream of the Ensembl published start site (http://www.ensembl.org/index.html), and gave rise to a distinct unique first exon of 60 bp termed exon 1A. The liver specific transcripts were termed P2 and P3. P2 comprises exon 1B, 2 and 3. Exon 1B (previously exon 1) is 67 bp longer than the original exon 1 at the 5′ end resulting in a 318 bp exon. The P3 transcript contains exon 2A which is a 145 bp novel exon located several Kb downstream from exon 2, and has been termed exon 2A. This exon is spliced directly onto Ensembl exon 3, and is the only transcript that does not contain exon 2 (Figure.1c). All these exons conform to the GT:AG splice site rule (Table 2). Comparison of the rat, mouse and human PPARα 5′UTR’s revealed that the previously unidentified exon 1A, found in the rat 5′UTR was homologous to the mouse exon 1A and human exon A. The 5′ end of this exon is longer in the human and mouse corresponding exons, but all share the same 3′ exon boundary. Exon 1B in rat is longer at the 5′end than previously reported making it more homologous with the mouse and human corresponding exons. Rat exon 2A identified by 5′RACE had no corresponding exon in the mouse 5′UTR but was present in the human 5′UTR. (Figure 1d).

**Table 2:** Alternative PPARα 5′UTR exon/intron junctions conform to the GT-AG splice site rule.

Transcript	Exon (bp)	5′ Donor Splice Site Consensus5′-gtaagt-3′	3′ Acceptor Splice Site Consensus5′-PyPyPyPyPyPyncag-3′	Exon (bp)
**Adipose P1**	1A (60)	CCTCAGgtgccc	cgttctatagCCAAGA	2 (87)
	2 (87)	TCACAGgtaaga	cctcctacagATTGGT	3 (247)
**Liver P2**	1B (318)	GGCGAGgtaact	cgttctatagCCAAGA	2 (87)
	2 (87)	TCACAGgtaaga	cctcctacagATTGGT	3 (247)
**Liver P3**	2A (145)	CTTCTGgtaggt	cctcctacagATTGGT	3 (247)

5′ (donor) and 3′ (acceptor) intron splice site consensus sequences are indicated. The sequences at P1, P2 and P3 transcript exon/intron boundaries are shown. Splice site sequences within introns are shown in lowercase; Exons are shown in capitals, and sizes of exons are given. All transcript exon boundaries conform to the GT-AG splice site rule.

### The P2 Promoter has Highest Activity in Liver HepG2 Cells

**Figure 2:**
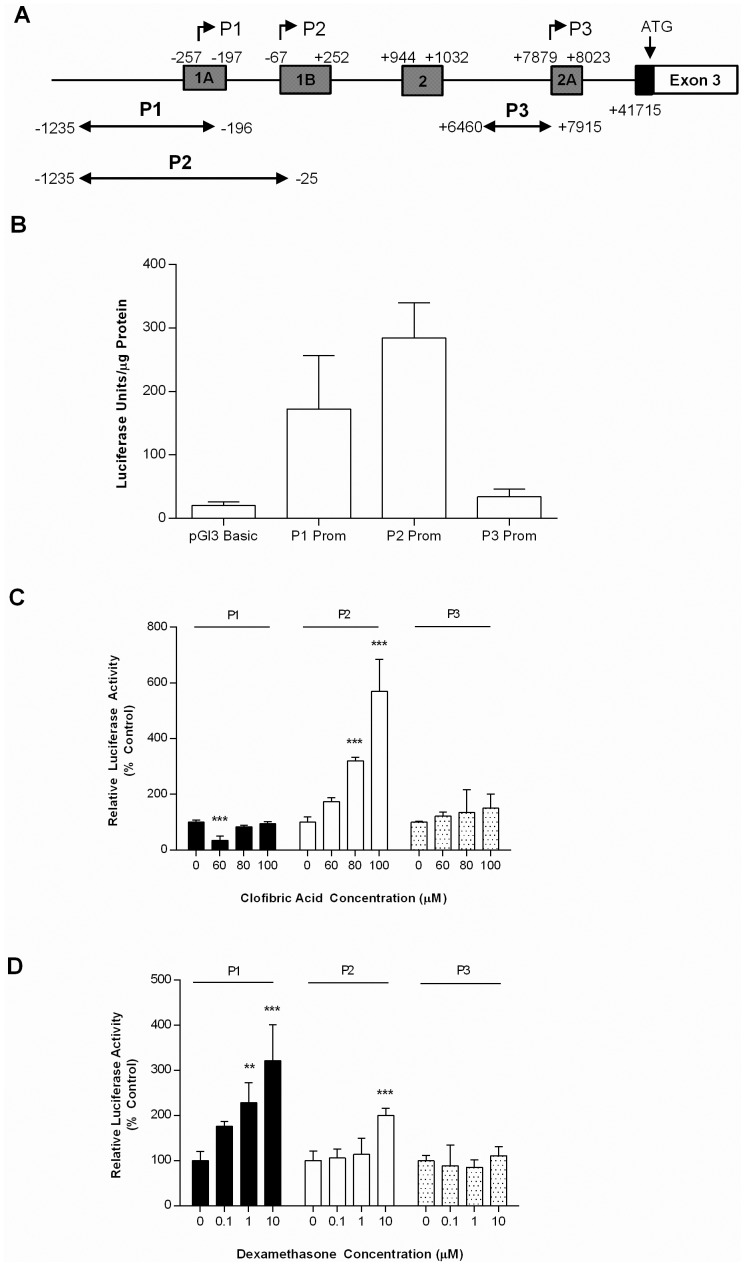
P1 and P2 promoters are active in HepG2 cells. A) Schematic diagram showing the relative locations of PPARα P1, P2 and P3 cloned promoter regions and their positioning relative to Ensembl transcription start site and 5′UTR exons. B) PPARα P1, P2 and P3 promoter constructs (P1, P2 and P3 Prom 1 µg) and an empty control vector (pGL3Basic 1 µg) were transfected into HepG2 cells and promoter activity assessed 24 hrs later. C and D) Regulation of PPARα promoter activity by clofibric acid and dexamethasone. P2 and P3 promoter constructs were transfected into HepG2 cells and treated for 24 hrs with vehicle control or an increasing concentration of dexamethasone (0, 0.1, 1 or 10 µM) or clofibric acid (60, 80, 100 µM). All values represent the mean of 6 independent experiments ±SEM.). Statistical comparisons of luciferase activity between treatments relative to the untreated control were determined by ANOVA followed by Bonferroni post hoc analysis. (* p<0.05, ** p<0.001, *** p<0.001).

To investigate whether the alternative PPARα exons are associated with promoter activity, the 5′ region immediately upstream of the P1, P2 and P3 transcription start sites (from approx −1.5 kb to +50 bp) were cloned into the reporter vector pGl3basic and transfected into HepG2 cells. This resulted in two partially overlapping promoter regions for P1 and P2 and a distinct downstream promoter for P3 (Figure. 2a). Both P1 and P2 promoters were active in HepG2 cells with P2 having the highest promoter activity. The P3 promoter region showed very low levels of activity, similar to that of the promoter-less pGL3 Basic vector (Figure 2b).

To determine whether the individual PPARα promoters are differentially regulated, the PPARα promoter-pGL3 luciferase reporter constructs (P1, P2 and P3) were transfected into HepG2 cells and treated for 24 hrs with clofibric acid and dexamethasone at a range of concentrations which have previously been reported to induce PPARα expression [12], [15]. Clofibric acid treatment repressed P1 promoter activity at 60 µM (2.8 fold p = 0.001), but induced P2 promoter activity at 80 µM (3.19 fold p = 0.001) and 100 µM (5.7 fold p = 0.001)(Figure. 2C). Both P1 and P2 promoter activity was increased in the presence of dexamethasone. A significant increase in P1 promoter activity at both 1 µM dexamethasone (2.2 fold p = 0.01) and 10 µM dexamethasone (3.22 fold p = 0.001) was observed. With P2 a significant rise in promoter activity was observed with 10 µM dexamethasone (2 fold p = 0.001)(Figure. 2D). There was no effect of either dexamethasone or clofibric acid on P3 promoter activity.

### Leptin Treatment Induces Transcription from the P2 but not the P1 or P3 Promoter

**Figure 3:**
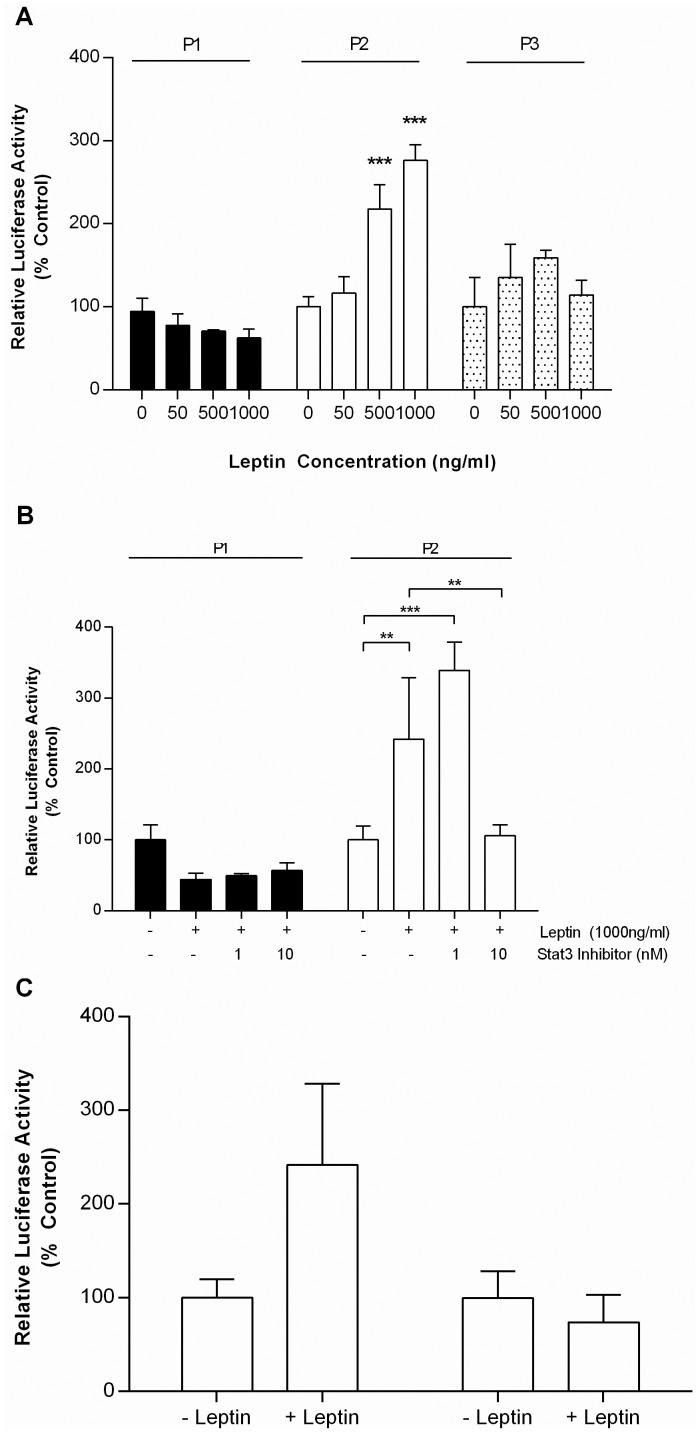
Leptin activates the PPARα P2 Promoter in HepG2 Cells P1, P2 and P3 promoter constructs were transfected into HepG2 cells and treated for 24 hrs with a vehicle control or an increasing concentration of leptin (0,50, 500, 1000 ng/ml). B) P1 and P2 promoter constructs were transfected into HepG2 cells and treated for 24 hrs with leptin (1000 ng/ml) and an increasing concentration of the STAT3 inhibitor PpYLKTK-mts (0,1,10 nM). C) Mutation of the Sp1 site blocks leptin activation of P2 promoter activity. P2 (P2-pGL3) and P2 promoter construct containing the mutated Sp1 response element (SP1M EcoRI-pGL3) was transfected into HepG2 cells and treated with leptin (1000 ng/ml) for 24 hrs. All values represent the mean of 6 independent experiments ±SEM. Statistical comparisons of luciferase activity between treatments relative to the untreated control were determined by ANOVA followed by Bonferroni post hoc analysis. (* p<0.05, ** p<0.001, *** p<0.001).

To determine whether leptin could induce transcription from the alternative PPARα promoters, HepG2 cells were transfected with the P1, P2 and P3 promoter constructs and treated with increasing concentrations of leptin for 24 hrs. We found that P2 promoter activity was significantly increased in the presence of 500 ng/ml and 1000 ng/ml leptin. In contrast, leptin had no effect on either P1 or P3 promoter activity (Figure. 3a). As leptin has been shown to modulate transcription through a STAT3 signalling pathway [33] we used the highly selective STAT3 inhibitor (PpYLKTK-mts) [34] to test the role of STAT3 in the induction of P2 transcription by leptin. We found that while the STAT3 inhibitor had no effect on P1 promoter activity, the STAT3 inhibitor blocked leptin activation of P2 promoter activity at 10 nM (Figure. 3b).

Leptin modulation of transcription via STAT3 has been shown to occur either directly through the binding of STAT3 to its response element in the promoter of a gene or indirectly through a STAT3/Sp1 co-operative mechanism [35]. As the PPARα P2 promoter lacks a STAT3 binding site but contains a Sp1 site which is located within the region unique to the P2 promoter, we next investigated whether this Sp1 site was essential for leptin induction of P2 transcription. The Sp1 site was mutated from GGCGGG to the EcoR1 recognition site GAATTC. The wild type and mutated promoter constructs were then transfected into HepG2 cells and treated with 1000 ng/ml of leptin for 24 hrs. We found that the P2 promoter containing the mutated Sp1 site no longer responded to leptin, suggesting that this Sp1 site is essential for leptin activation of PPARα transcription (Figure.3C).

### Differential Regulation of Alternative PPARa Transcripts by Neonatal Leptin

**Figure 4:**
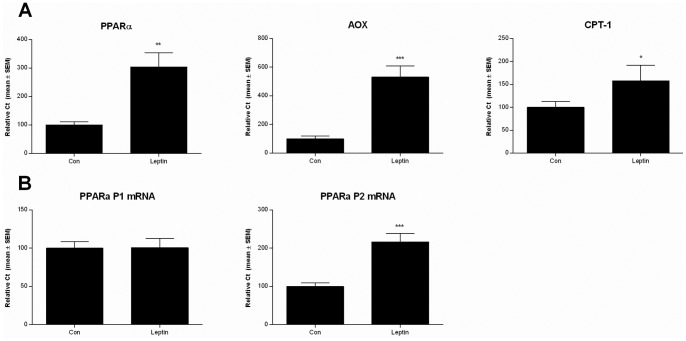
Neonatal leptin treatment leads to a persistent increase in PPARα P2 transcription. **A**) Neonatal leptin treatment induces a persistent increase in total PPARα transcripts, AOX and CPT-1 mRNA expression in retroperitoneal adipose. Values represent the mean ± SEM relative to the saline treated control group. Statistical comparisons between the control (Con) and neonatal leptin treated (Leptin) groups were made using a Students unpaired t test. B) The expression of the P2 but not the P1 PPARα transcript is significantly altered by neonatal leptin treatment. Values represent the mean ±S EM relative to the saline treated control group. Statistical comparisons between the control (Con) and leptin treated (Leptin) groups were made using Students unpaired t test. (* p<0.05, ** p<0.001, *** p<0.001).

As there is evidence that hepatic PPARα gene transcription can be programmed by environmental factors in early life including leptin, we next investigated whether neonatal leptin treatment induced a persistent increase in PPARα expression in adipose tissue and whether the P1 and P2 transcripts were differentially affected. The expression of PPARα together with its target genes AOX and CPT-1 were examined in adipose tissue from PN170 rats treated with either saline or leptin (2.5 µg/g/day) by subcutaneous injection for 10 days from PN3-13. This dosage of leptin was used, as similar levels have been reported previously to induce leptin receptor signaling, alter neuropeptide expression [36], [37] and reverse the metabolic features induced by maternal under nutrition. To analyze PPARα expression, primers were designed to anneal to the coding region of PPARα, a region common to all isoforms of PPARα, in order to measure total PPARα transcript levels, and to the specific P1 and P2 transcripts. Neonatal leptin administration led to an increase in total PPARα, AOX and CPT-1 mRNA in adipose tissue from D170 old rats compared to saline treated controls. However there was no effect of leptin treatment on the expression of the P1 transcript (Figure. 4b), while neonatal leptin treatment significantly increased P2 specific transcripts in adipose tissue.

**Figure 5:**
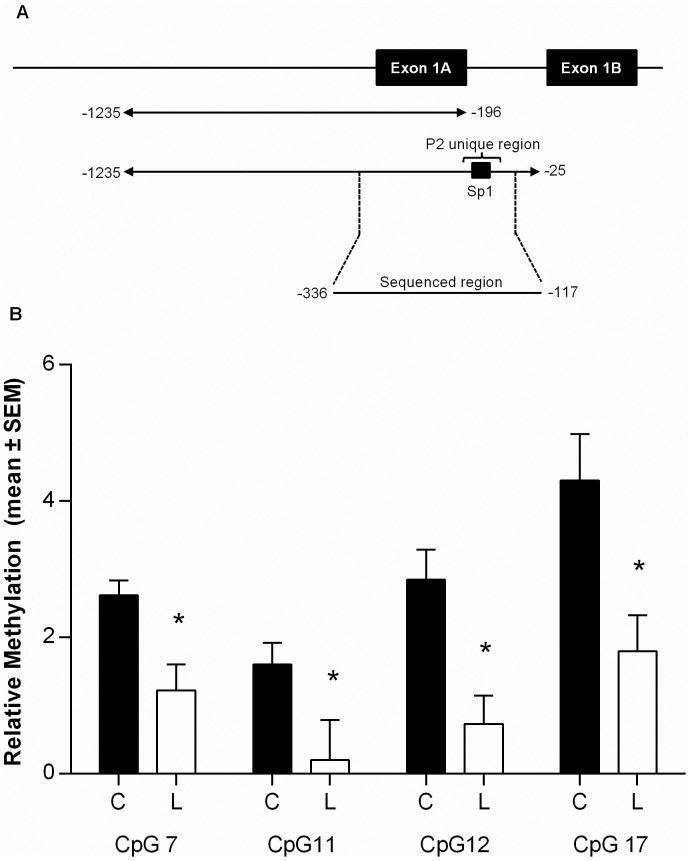
Pyrosequencing analysis of the PPARα promoter in adipose tissue of saline or leptin treated adult female rats. A) Schematic diagram showing the location of the CpGs sequenced. All exon positions are indicated relative to the Ensembl transcription start site B) Pyrosequencing analysis of CpGs within the PPARα promoter. Values represent mean methylation levels ±SEM (n = 8/group).Only CpG sites where a significant difference in methylation between the saline and leptin treated groups are shown. Saline treated, black bars (C), leptin treated white bars (L). Significant differences in DNA methylation between saline and leptin treated groups was determined using a Students unpaired t-test where *p<0.05.

To determine whether this persistent increase in transcription from the P2 promoter in response to neonatal leptin treatment was due to altered DNA methylation, sodium bisulfite pyrosequencing was performed using genomic DNA extracted from adipose tissue from neonatal saline and leptin treated adult female rats. The analysis of the region (−336 to −117 bp) immediately upstream of the PPARα P2 transcription start site (TSS) which contains the Sp1 response element in the P2 unique region showed that all CpGs within this region had a methylation level of below 10% regardless of treatment (Figure 5.), although differences in methylation were observed between the leptin and saline treated offspring at CpGs 7,11,12 and 17 (CpG 7 (2.6% to 1.2%,), CpG 11 (1.6% to 0.2% ), CpG 12 (2.85% to 0.73%) and CpG 17 (4.3% to 1.8%,).

## Discussion

The genomic organisation of the human and mouse PPARα genes indicate that PPARα, like other nuclear receptors, contains multiple 5′UTR variants and promoter regions [27]. Previously rat PPARα was not known to have any mRNA variants and only three exons (exons 1, 2 and 3) were found to encode the 5′ UTR. In this study, 5′ RACE analysis revealed three PPARα mRNA variants, with 2 transcripts identified in liver (P2 and P3) and one in adipose (P1). These transcripts differed from each other at the 5′ end, and comparisons to the genomic sequence showed that they had different transcription start sites owing to the presence of unique first exons. Five exons were found to encode the PPARα 5′UTR, Exons 1A and 2A were novel, one exon was a modified version of exon 1 (now termed 1B), and the remainder were exactly as identified previously [26]. The identification of the additional 5′UTR first exons in the rat PPARα promoter increases the homology between the rat, mouse and human PPARα promoter structures. For example, the 5′ extended exon 1B in the rat P2 transcript and the novel exon 1A in the rat P1 transcript are both present in the human and mouse 5′UTR. In addition, the rat P1 and P2 PPARα transcripts are very similar with those identified in the mouse, the rat P2 transcript being equivalent to the mouse variant 1 and the newly identified rat P1 transcript being similar to the mouse variant 2.

Since the 5′ UTR can modulate RNA stability[38]–[40], translation efficiencies [41], [42] as well as subcellular localisation [43], [44], the use of alternative promoters to regulate the expression of untranslated first exons may add a further layer of control to the regulation of PPARα expression. For example, the presence of a long 5′UTR, high GC content, secondary structure, uATGs and uORFs are all associated with reduced translational efficiency of the main ORF [45]. The relatively high GC content of the 5′UTR was fairly consistent between the three PPARα alternative transcripts, but the length of the 5′UTR varied from 184 bp for P3, to 444 bp for P2. This difference in length was reflected by differences in the minimum free energies of the transcripts calculated using Zucker RNA mfold 2.3 software which ranged from −74.65 Kcal/mol for P1, −169.75 Kcal/mol for P2 and −56.15 Kcal/mol for P3 [46]. Secondary structures within the 5′UTR with values of less than 30 Kcal/mol can be melted by the ribosome during the normal scanning process [47]. However, all three PPARα transcripts contain hairpins with stabilities of greater than this, indicating these transcripts may impede ribosomal movement and may be subjected to translational regulation.

The P1 and P2 transcripts did not contain ATG initiation codons in the sequence upstream of the previously reported ATG codon suggesting that they possess the same open reading frame as the previously reported transcript. The P3 transcript, however, contains four uATGs, 3 have adequate Kozak consensus sequences [47], but are followed by a termination codon. However, one ATG is in frame with the downstream ATG, and thus has the potential to produce a protein with a 30aa extended N terminal. PPARγ2 has an extended N terminal of 30aa and 28aa in mice and humans, respectively, compared to the predominant PPARγ1 protein. Experiments have shown that the PPARγ2 N terminal extension comprises an N terminal ligand independent activation domain that mediates 5–6 times the activation of PPARγ1 under ligand depleted conditions [48]. However, further studies are needed to determine whether the PPARα P3 transcripts contain a functional extended protein.

The analysis of the sequence upstream of the transcription start sites of the P1, P2 and P3 sites revealed that the P1/P2 promoters possess the characteristics of typical GC rich promoters common to nuclear hormone receptors such as the absence of TATA elements and the presence of CpG islands containing multiple Sp1 response elements. The P3 promoter, in contrast, did not contain any CpG islands or Sp1 response elements. Moreover, unlike the sequences upstream of the P1 and P2 TSS, which gave rise to high levels of promoter activity in HepG2 cells, the sequence upstream of the P3 start site was not active in HepG2 cells. This low activity may indicate that important regulatory elements outside the region cloned in this study are required for P3 expression or that the promoter is inactive in the absence of stimulatory factors that are not present in liver cell line HepG2.

It has been reported previously that PPARα transcription is induced by CFA [15], dexamethasone [12] and leptin [49]. Interestingly, the response of the adipose specific (P1) and liver specific (P2) promoters to these treatments differed. Both P1 and P2 promoters were up-regulated by dexamethasone, suggesting that glucocorticoids modulate PPARα expression through a sequence shared by the P1 and P2 promoters. Previous experiments have shown that GR can directly regulate PPARα expression [50] although the precise sequence was not identified. Matinspector analysis (www.genomatix.de) of the promoter region of PPARα did not reveal any glucocorticoid response elements, but a putative NF-1 binding site was identified in the sequence shared by both P1 and P2. NF-1 is a transcription factor that has been shown to mediate GR responsiveness [51]. In contrast, P1 and P2 promoters were differentially regulated by clofibric acid, a PPARα agonist and leptin, suggesting that leptin and clofibric acid mediate their effects through a sequence(s) that are unique to the P2 promoter. Autoregulation of gene expression is commonly found in nuclear receptors and ligands of PPARα have previously been reported to activate PPARα expression at the transcriptional level by binding to either a PPRE or DR1 motif [24], the latter of which is present within the unique region of the P2 promoter. Leptin has been suggested to regulate gene expression through the activation of Stat3 via a JAK signalling pathway [52]–[54], or in a promoter which lack a STAT3 response element, through a Stat3-Sp1 co-operative mechanism whereby Stat3 phosphorylates Sp1 which, in turn, facilitates Sp1 binding to its response elements [55]. The mechanism of leptin induction of P2 transcription involved both Stat3 and an Sp1 response element present in the sequence that is unique to the P2 promoter.

Leptin plays a critical role in maintaining energy balance [56], [57] and also has an emerging role in growth and development [58]. Adipogenesis is associated with a marked elevation in serum leptin concentration which occurs between 4 to 10 days after birth in mice [59], while in rats, peak leptin concentration occurs at about 10 days after birth [60]. This increase in leptin has been shown to play a crucial neurotrophic role in the development of projections from the arcuate nucleus of the hypothalamus, which regulates food intake and adiposity [36], [61]. Several reports indicate that the neonatal leptin surge is disturbed in its timing and/or magnitude by maternal undernutrition [61], [62]. Moreover neonatal leptin administration from PN3–13 has been shown to reverse many of the features of metabolic programming induced by maternal undernutrition by slowing neonatal weight gain, normalizing caloric intake, locomotor activity, body weight and fat mass in adult offspring of undernourished mothers fed a HF diet [23]. The mechanism by which neonatal leptin treatment induces persistent changes in the regulation of fat mass was presumed to involve an effect on hypothalamic neurogenesis. Our findings however show that neonatal leptin administration induces a persistent increase in PPARα mRNA expression and its target genes AOX and CPT-1 in adipose tissue, suggesting that leptin may also have peripheral metabolic effects on adipose tissue. The increase in PPARα mRNA is consistent with the previous reports that hyperleptineamia induces PPARα expression in adipose tissue. Although interestingly while a transient increase in PPARα expression was observed in response to hyperleptinemia which disappeared when levels of leptin returned to normal, neonatal leptin treatment induced a stable increase in PPARα expression in adipose tissue which persisted into adulthood. This difference in the duration of the response to leptin may reflect the timing of the exposure during the life-course, as there is now growing evidence that early life exposures can induce long term effects on the metabolism and physiology of the offspring via epigenetic processes [63]. Consistent with the induction of P2, but not P1, promoter activity in cell culture experiments, neonatal leptin treatment differentially-regulated transcription from the PPARα P1 and P2 promoters in adipose tissue by persistently inducing P2, but not P1, transcription. A decrease in methylation at four CpGs within the P2 promoter was seen in the neonatal leptin treated compared to the saline treated animals. However given the low levels of methylation in this region, it is unclear whether such differences in methylation would mediate a switch in promoter usage although there is precedent for differences in methylation at low levels altering gene function [64].

In conclusion we show that the rat PPARα gene has multiple transcripts, which are expressed in a tissue specific manner and are differentially regulated by leptin. Moreover neonatal leptin exposure induces a persistent change in adipose tissue gene expression through specific activation of an otherwise quiescent PPARα promoter. It is possible that leptin may have similar effects in other species. For example, human PPARα also has multiple promoters and hence it is possible that long term modulation of PPARα activity, and hence lipid metabolism, by promoter switching as a result of leptin exposure in early life may also occur in humans. If the changes to gene expression in adipose tissue reported in the present study were to occur in humans, it may be expected that those who experience higher leptin exposure in early life would have greater capacity to meet the metabolic challenge of a high calorie diet after weaning, while a lower leptin exposure would result in a reduced capacity to regulate fatty acid deposition in adipose tissue. This may explain, at least in part, the observation that low umbilical cord blood leptin levels are associated with rapid postnatal weight gain [65]. One implication is that leptin exposure during specific periods in development may influence future risk of obesity.
